# Mechanisms of the TGF-β1/Smad3-signaling pathway in gender differences in alcoholic liver fibrosis

**DOI:** 10.1186/s12576-024-00901-y

**Published:** 2024-02-26

**Authors:** Xiaomin Hong, Sanqiang Li, Renli Luo, Mengli Yang, Junfei Wu, Shuning Chen, Siyu Zhu

**Affiliations:** 1 The Molecular Medicine Key Laboratory of Liver Injury and Repair, College of Basic Medicine and Forensic Medicine, Henan University of Science and Technology, Luoyang, 471003 People’s Republic of China; 2 Henan Center for Engineering and Technology Research On Prevention and Treatment of Liver Diseases, Luoyang, 471003 People’s Republic of China; 3 Pharmaceutical Science, Monash University, Melbourne, Australia

**Keywords:** Alcoholic liver fibrosis, Gender differences, Estradiol, Testosterone, TGF-β1/Smad3-signaling pathway

## Abstract

The TGF-β1/Smad3-signaling pathway and gender differences were investigated in alcoholic liver fibrosis. Mice were divided into female normal, female model, male normal, and male model groups. Liver injury and fibrosis were assessed using histopathology and serology. Western blotting was performed to analyze the expression of relevant factors. HSC-T6 cells were divided into estradiol + saline, estradiol + ethanol, testosterone + saline, and testosterone + ethanol groups, and similar assessments were conducted in vitro. Compared with the female model group, the male model group exhibited significantly increased GPT, GOT, TNF-α, IL-6, and testosterone levels, fibrosis rate, and TGF-β1, Smad3, and PCNA expression, and significantly decreased estradiol levels and Caspase-3 expression. The apoptosis rate was higher in the estradiol + ethanol group than in the testosterone + ethanol group, although the testosterone + ethanol group exhibited significantly increased TNF-α, IL-6, Collagen-I, α-SMA, TGF-β1, Smad3, and PCNA expression, and significantly decreased Caspase-3 expression. Alcoholic liver fibrosis showed significant gender differences associated with the TGF-β1/Smad3-signaling pathway.

## Background

Alcoholic liver fibrosis results from the impact of ethanol and its metabolites on the liver, causing excessive proliferation and abnormal deposition of extracellular matrix (ECM) in liver tissue, with hepatic stellate cells (HSCs) playing a pivotal role [1]. Epidemiological data have revealed a growing population of drinkers, with alcoholic liver disease accounting for 35.5% of chronic liver disease cases in mainland China in 2018. Moreover, there are significant gender differences in alcoholic liver disease, as indicated by the 2022 China Health Statistics Yearbook, which reports a prevalence ratio of approximately 2.7:1.0 between men and women in relation to alcoholic liver fibrosis. These differences may be associated with androgenic hormone levels [2].

In recent years, research has focused on the TGF-β1/Smad3-signaling pathway, which plays a crucial role in the development of alcoholic liver fibrosis. Multiple studies have found that estrogen functions as a regulator of various signaling pathways [3]. However, the relationship between gender differences and the TGF-β1/Smad3-signaling pathway in alcoholic liver fibrosis isremains unclear. To address this knowledge gap, we conducted in vitro experiments using HSC-T6 cells stimulated with physiologically relevant levels of androgen and cultured in an ethanol-containing medium to observe HSC activation. In addition, in vivo experiments were performed by inducing ethanol-induced liver fibrosis in female and male mice, providing novel insights into this field of research.

## Methods

### Cell culture and treatment

Mouse hepatic stellate cells (HSC-T6) were obtained from Zhongqiao Xinzhou Biotechnology Co., Ltd. (Shanghai, China). The cells were cultured in high-sugar medium (Dulbecco’s Modified Eagle’s Medium; Biosharp Biotechnology Co. Ltd., Zhejiang, China) supplemented with 10% fetal bovine serum and penicillin–streptomycin double antibodies. The culture was maintained at 37 °C under 5% CO_2_ and saturated humidity. HSC-T6 cells were divided into four groups: estradiol + saline, estradiol + ethanol, testosterone + saline, and testosterone + ethanol. The cells in the estradiol + saline and testosterone + saline groups were cultured under the same conditions with a physiological concentration (10^−7^ mol·L^−1^) of estradiol for 48 h and then with a culture medium containing saline for another 48 h. The cells in the estradiol + ethanol and testosterone + ethanol groups were cultured similar to estradiol, but then with a culture medium containing 100 mmol·L^−1^ alcohol for 48 h.

### Proliferation activity of cells by based on the MTT assay

HSC activated-T6 cells in the logarithmic growth phase were inoculatedseeded in 96-well plates and incubated at 37 °C in a constant temperature CO_2_ incubator at 37℃ for 20 h, supernatant. The culture medium was discarded, replaced with 200 μL of culture solution containing estradiol different concentrations (10^−3^, 10^−4^, 10^−5^, 10^−6^, 10^−7^, 10^−8^, 10^−9^, and 10^−10^ mol·L^−1^) of estradiol and testosterone (10^–3^, 10^–4^, 10^–5^, 10^–6^, 10^–7^, 10^–8^, 10^–9^, 10^–10^) mol·L^−1^, respectively, were added to each well for 48 h, and Each concentration was tested in six groups were set up in each. After that 48 h of incubation, 20 μL of MTT (5 g·L^−1^) MTT was added, purchased from Solebro Technology Co. Then was added to each well, followed by the addition of 200 μL of DMSO was added to each well, dimethyl sulfoxide purchased from Damao Chemical Reagent Factory (Tianjin, China). The plate was protected from light, shaken for 10 min with an oscillator, and the absorbance values at 570 nm were measured using an enzyme marker within 10 min to calculate the cell proliferation rates.

### Detection of apoptosis through flow cytometry

HSC-T6 cells were collected and resuspended in 250 μL of diluted binding buffer (binding buffer:deionized water ratio = 1:4). The cells were adjusted to a concentration of 1 × 10^6^ mL^−1^ with an Annexin V-FITC/propanol iodide (PI) double-staining apoptosis assay kit (Biosharp Biotechnology Ltd., Guangzhou, China) at room temperature (20 °C–25 °C) while protected from the light. The cells were stained with 5 µL Annexin V-FITC for 10 min at room temperature (20–25 °C) and 10 µL PI for 5 min prior to loading and subsequently analyzed using flow cytometry.

### Detection of cellular TNF-α and IL-6 levels

HSC-T6 cells in the logarithmic growth phase were cultured in six-well plates and divided into four groups: estradiol + saline, estradiol + alcohol, testosterone + saline, and testosterone + alcohol. When the cell density reached 60%, the cells in the estradiol + saline and testosterone + saline groups were incubated with physiological concentrations of estradiol and testosterone for 48 h, followed by a culture medium containing saline for another 48 h. The cells in the estradiol + alcohol and testosterone + alcohol groups were incubated with physiological concentrations of estradiol and testosterone for 48 h, followed by a culture medium containing alcohol for another 48 h. The supernatants from the six-well plates were then added to an enzyme-free sterile EP tube and centrifuged in a low-temperature centrifuge at 2000 rpm for 20 min. The supernatant was collected, and the liquid was diluted with 1 × phosphate-buffered saline and centrifuged again in the same centrifuge at 2000 rpm for 20 min. The supernatant was collected and centrifuged again under the same conditions to obtain the cellular TNF-α and IL-6 levels. Respective kits were purchased from Yubo Biologicals (Shanghai, China) and used following the manufacturer’s instructions.

### Establishment of alcoholic liver fibrosis mouse model

Male and female C57BL/6N mice (specific pathogen-free grade; SCXK20210006), aged 6–8 weeks and weighing 22 ± 2 g, were obtained from Beijing Vitality Animal Technology Co. The mice were housed in the animal room of the College of Basic Medicine and Forensic Medicine of Henan University of Science and Technology under controlled conditions (temperature: 18℃–25℃; light period: 12 h light/12 h dark). After acclimatization feeding for 1 week, the mice were randomly divided into four groups: male normal, male model, female normal, and female model, with 15 mice in each group. The normal groups were fed with TP4030C Lieber–DeCarli liquid diet (47% fat, 18% protein, and 35% sugar) for 8 weeks, whereas the model groups were fed with TP4030A Lieber–DeCarli liquid diet (35% fat, 18% protein, 19% sugar, and 28% ethanol) for 8 weeks combined with gavage-administered 31.5% ethanol at a dose of 5 g·kg^−1^ twice a week. The diets and ethanol were purchased from Trophic Feeds Ltd. (Nantong, China).

### Detection of serum GPT, GOT, estradiol, testosterone, TNF-α, and IL-6 levels

Blood samples were collected, allowed to naturally clot at room temperature for 10–20 min, and centrifuged at 3000 rpm for 10 min. After centrifugation, the supernatant was collected in a new EP tube and placed on ice, and GPT, GOT, estradiol (E_2_), testosterone (T), TNF-α, and IL-6 levels were detected using kits purchased from Yubo Bio (Shanghai, China) according to the manufacturer’s instructions.

### Histopathological analysis of liver tissue

Liver tissues were fixed in EP tubes containing 4% paraformaldehyde for 24–48 h, followed by sequential ethanol gradient dehydration, embedding, sectioning, dewaxing, and gradient ethanol hydration. Subsequently, hematoxylin, and eosin HE, Sirius Scarlet, glycogen, and Oil Red O staining were performed according to the respective kit instructions (all purchased from Solebro Technology, Beijing, China). The stained tissues were observed and photographed using an Axio Observer3 fluorescence microscope.

### Detection of collagen-I, α-SMA, TGF-β1, Smad3, PCNA, and Caspase-3 expression levels through immunoblotting

In vitro experiments were conducted using HSC-T6 cells in the logarithmic growth stage. The cells were seeded in six-well plates and divided into four groups: estradiol + saline, estradiol + alcohol, testosterone + saline, and testosterone + alcohol. When the cell density reached 60% in the wells, the estradiol + saline and testosterone + saline groups were incubated with physiological concentrations of E_2_ and T for 48 h, followed by incubation with culture medium containing saline for another 48 h. Similarly, the estradiol + alcohol and testosterone + alcohol groups were incubated with physiological concentrations of E_2_ and T for 48 h, followed by alcohol-containing culture medium for an additional 48 h. After the incubation period, cell protein extraction was performed.

For in vivo experiments, liver tissues were collected at the end of 8 weeks. The tissues were mixed with lysate in proportion and placed on ice. The tissue was ground, and the ground homogenate was transferred to EP tubes and centrifuged at 12,000 rpm for 20 min at 4℃. The supernatant was collected to determine the protein concentration and calculate the appropriate protein loading volume. Subsequently, the proteins were loaded onto a gel, electrophoresed, transferred to a membrane, and blocked with 5% skim milk powder at room temperature for 1 h. The membrane was washed three times with PBS with Tween (PBST), and primary antibodies (Collagen-I, α-SMA, TGF-β1, Smad3, PCNA, and Caspase-3; all diluted 1:1000; Santa Cruz Biotechnology, USA) were added to the membranes and incubated overnight at 4℃. On the following day, the membranes were incubated with horseradish peroxidase-labeled goat or rabbit anti-mouse IgG polyclonal antibody (diluted 1:2000; ZhongShan JinQiao Biological Company, Beijing, China) at room temperature for 1 h. The membranes were washed again with PBST and exposed to an enhanced chemiluminescent chromogenic solution (Qiaoxing Trading Co.) The acquired images were analyzed using Analyzer software 4.0, and the results were normalized to β-actin.

### Data analysis

All experiments were repeated three times, and the experimental data were statistically analyzed using SPSS 25.0 software. All data satisfied normal distribution, and one-way ANOVA was used to compare the differences between multiple groups if the variances were aligned; if the variances were not aligned, the Brown–Forsythe test was used. a = 0.05 was chosen as the test level, and P < 0.05 was considered to be statistically different.

## Results

### MTT assay results

**Fig. 1:**
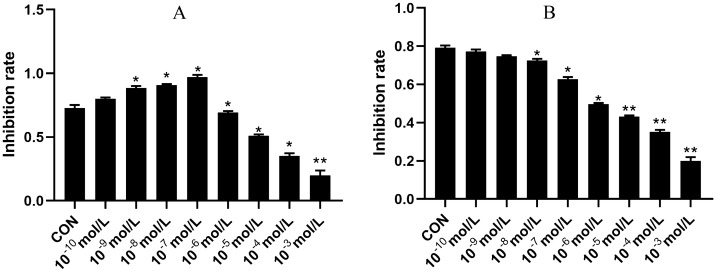
Effect of testosterone and estradiol on the proliferative capacity of HSCs. **a** Effect of testosterone on HSC proliferative capacity. **b** Effect of estradiol on HSC proliferative capacity. Comparisons made against the normal group: ^*^P < 0.05 or.^**^P < 0.01. Data are expressed as mean ± SD, *n* = 6

Following a 48 h incubation of activated HSC-T6 cells with physiological concentrations of E_2_ and T, the MTT colorimetric method was used to calculate the cell proliferation rate. E_2_ inhibited HSC growth within the concentration range 10^−9^–10^−7^ mol·L^−1^, with the most significant inhibition observed at 10^−7^ mol·L^−1^. Conversely, T promoted HSC growth, with the most significant promotion observed at 10^−7^ mol·L^−1^ (*P* < 0.05 or *P* < 0.01). Data are expressed as mean ± SD, *n* = 6 (Fig. 1).

### Flow cytometry results

**Fig. 2:**
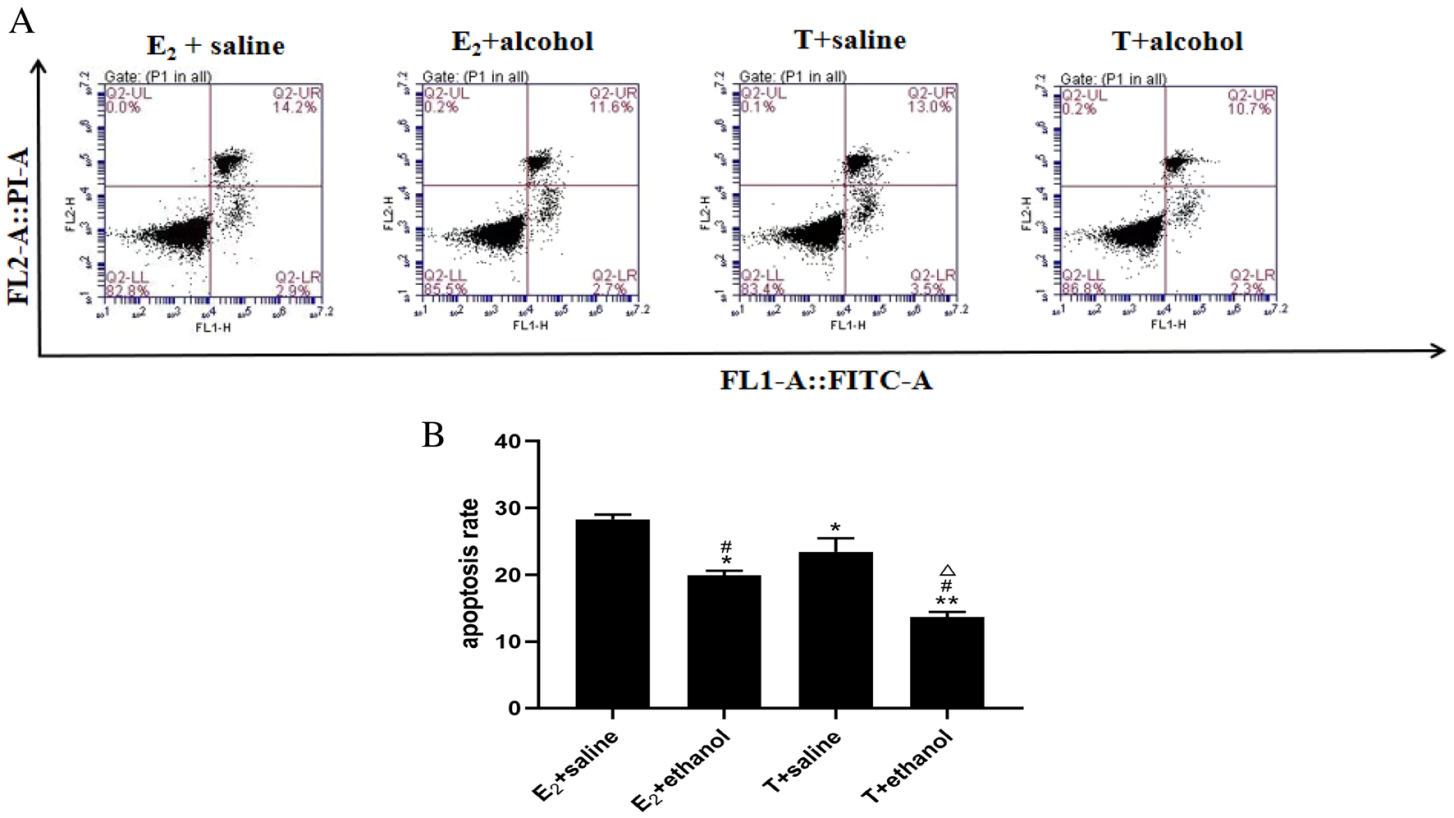
Effect of estradiol and testosterone on the apoptosis rate of activated HSCs detected via flow cytometry. **a** Apoptosis map of each group based on flow. **b** Apoptosis rate in each group (compared with the estradiol group, ^*^P < 0.05 or ^**^P < 0.01; compared with the testosterone group, ^#^*P* < 0.05 or ^##^*P* < 0.01; compared with the estradiol + ethanol group, ^△^*P* < 0.05 or ^△△^*P* < 0.01). Data are expressed as mean ± SD, *n* = 3

In both normal and model groups, activated HSC-T6 cells were first cultured with 10^−7^ mol·L^−1^ E_2_ and 10^−7^ mol·L^−1^ T for 48 h. In the normal groups, this was followed by culture with saline-containing medium for another 48 h, whereas in the model group, this was followed by stimulation with alcohol-containing medium for 48 h. Compared with the estradiol + saline group, the apoptosis rate was reduced in the testosterone + saline, estradiol + alcohol, and testosterone + alcohol groups. Furthermore, compared with the testosterone + saline group, the testosterone + alcohol group exhibited a significant reduction in apoptosis rate (*P* < 0.05 or *P* < 0.01). Data are expressed as mean ± SD, *n* = 3 (Fig. 2).

### Cellular TNF-α and IL-6 concentration detection results

**Fig. 3:**
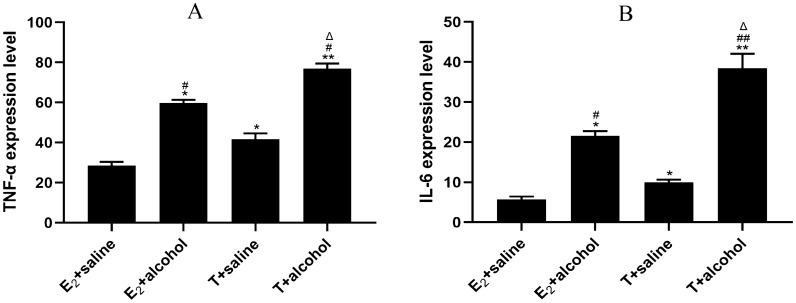
Concentrations of serum TNF-α and IL-6 in different groups of HSCs. **a** Concentration of TNF-α; **b** concentration of IL-6 (compared with the estradiol + saline group, ^*^P < 0.05 or ^**^P < 0.01; compared with the testosterone + saline group, ^#^*P* < 0.05 or ^##^*P* < 0.01; compared with the estradiol + alcohol group, ^△^*P* < 0.05 or ^△△^*P* < 0.01). Data are expressed as mean ± SD, *n* = 6

In both normal and model groups, activated HSC-T6 cells were first cultured with 10^−7^ mol·L^−1^ E_2_ and 10^−7^ mol·L^−1^ T for 48 h. In the normal groups, this was followed by culture with saline-containing medium for 48 h, whereas in the model groups, this was followed by stimulation with alcohol-containing medium for 48 h. Compared with the estradiol + saline and testosterone + alcohol groups, both the estradiol + saline and testosterone + alcohol groups exhibited increased concentrations of TNF-α and IL-6. Furthermore, the testosterone + alcohol group showed significantly higher concentrations of TNF-α and IL-6 compared with the estradiol + alcohol group (*P* < 0.05 or *P* < 0.01). Data are expressed as mean ± SD, *n* = 6 (Fig. 3).

### ***Serum GPT and GOT enzyme activity and E***_***2***_***, T, TNF-α, and IL-6 concentration results***

**Fig. 4:**
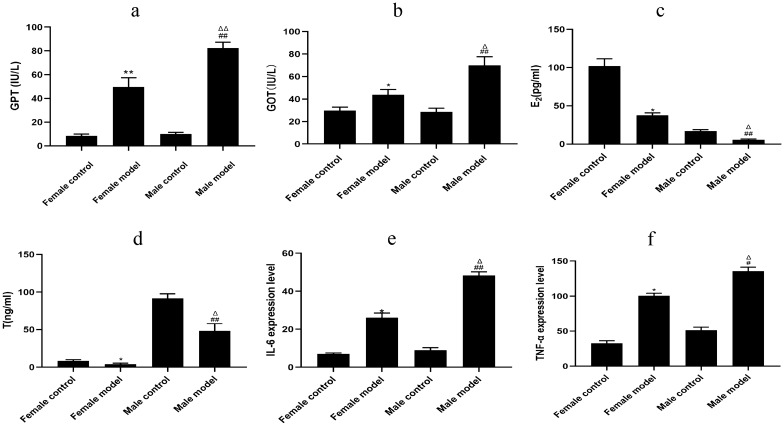
Enzyme activities and concentrations of serum GPT, GOT, E_2_, T, TNF-α, and IL-6 in different groups of mice. **a** GPT enzyme activity; **b** GOT enzyme activity; **c** E^2^ concentration; **d** T concentration; **e** IL-6 concentration; **f** TNF-α concentration [compared with the female normal (control) group, ^*^P < 0.05 or ^**^P < 0.01; compared with male control group, ^#^P < 0.05 or ^##^P < 0.01; compared with female model group, ^△^*P* < 0.05 or.^△△^*P* < 0.01]. Data are expressed as mean ± SD, *n* = 15

Following gavage with alcoholic liquid chow combined with 31.5% ethanol, the model group exhibited significantly higher GPT and GOT enzyme activities and significantly lower concentrations of E_2_, T, TNF-α, and IL-6 compared with the normal group. In addition, the male model group exhibited significantly higher serum GPT and GOT enzyme activities and significantly lower concentrations of E_2_, T, TNF-α, and IL-6 compared with the female model group (*P* < 0.05 or *P* < 0.01). Data are expressed as mean ± SD, *n* = 15 (Fig. 4).

### Liver histopathological results

**Fig. 5:**
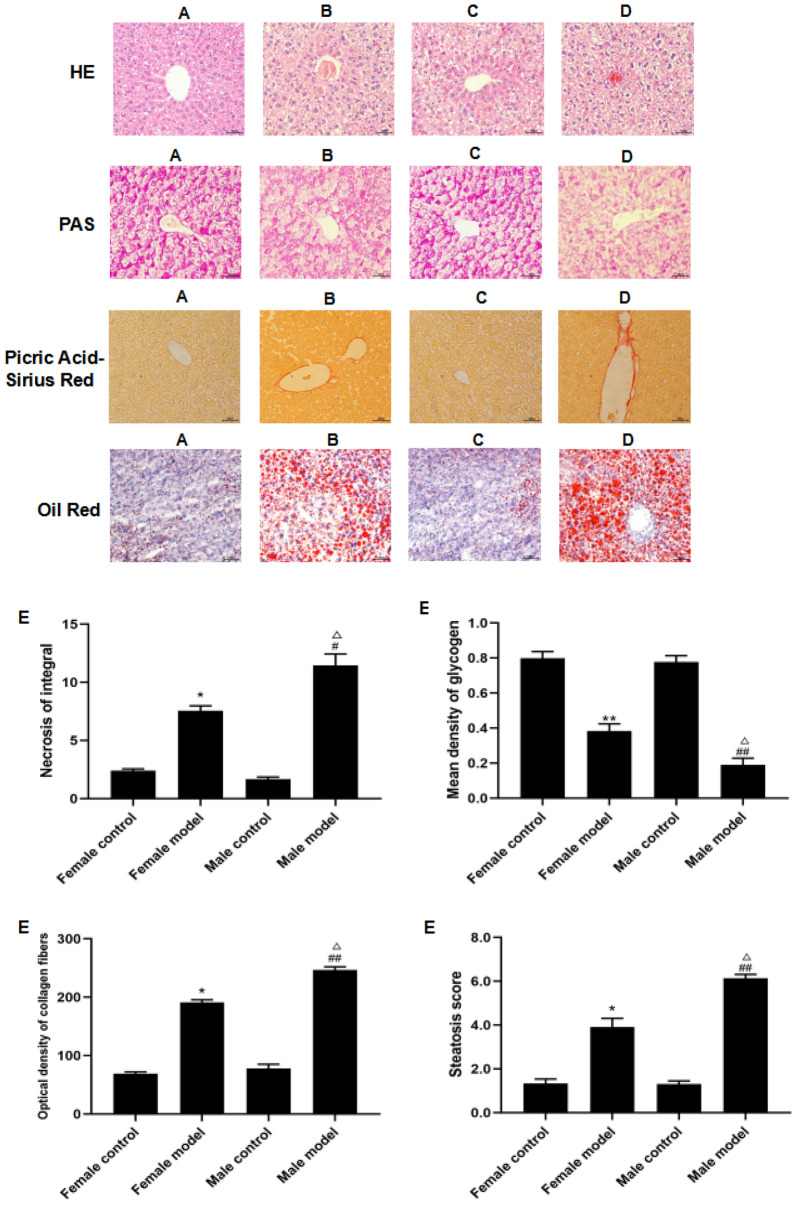
Changes in the liver tissues of mice in each group under a light microscope (× 200). **a** Female control group; **b** female model group; **c** male control group; **d** male model group; **e** liver necrosis, glycogen content, collagen fibrils, or lipid droplets [compared with the female normal (control) group, ^*^P < 0.05 or ^**^P < 0.01; compared with the male control group, ^#^*P* < 0.05 or ^##^*P* < 0.01; compared with the female model group, ^△^*P* < 0.05 or.^△△^*P* < 0.01]. Data are expressed as mean ± SD, *n* = 15

The model group, exposed to alcoholic liquid feed combined with 31.5% ethanol gavage, exhibited disrupted liver lobule structure, disordered arrangement of liver cords, infiltration of inflammatory cells, substantial deposition of collagen fibers in the portal area, a scarcity of purple–red glycogen particles, and an abundance of red lipid droplets. Compared with the female model group, the male model group displayed significantly higher HE necrosis scores, a greater area of collagen fibers, a reduced number of glycogen particles, and an increased number of lipid droplets (*P* < 0.05 or *P* < 0.01). Data are expressed as mean ± SD, *n* = 15 (Fig. 5).

### Protein immunoblotting results

**Fig. 6:**
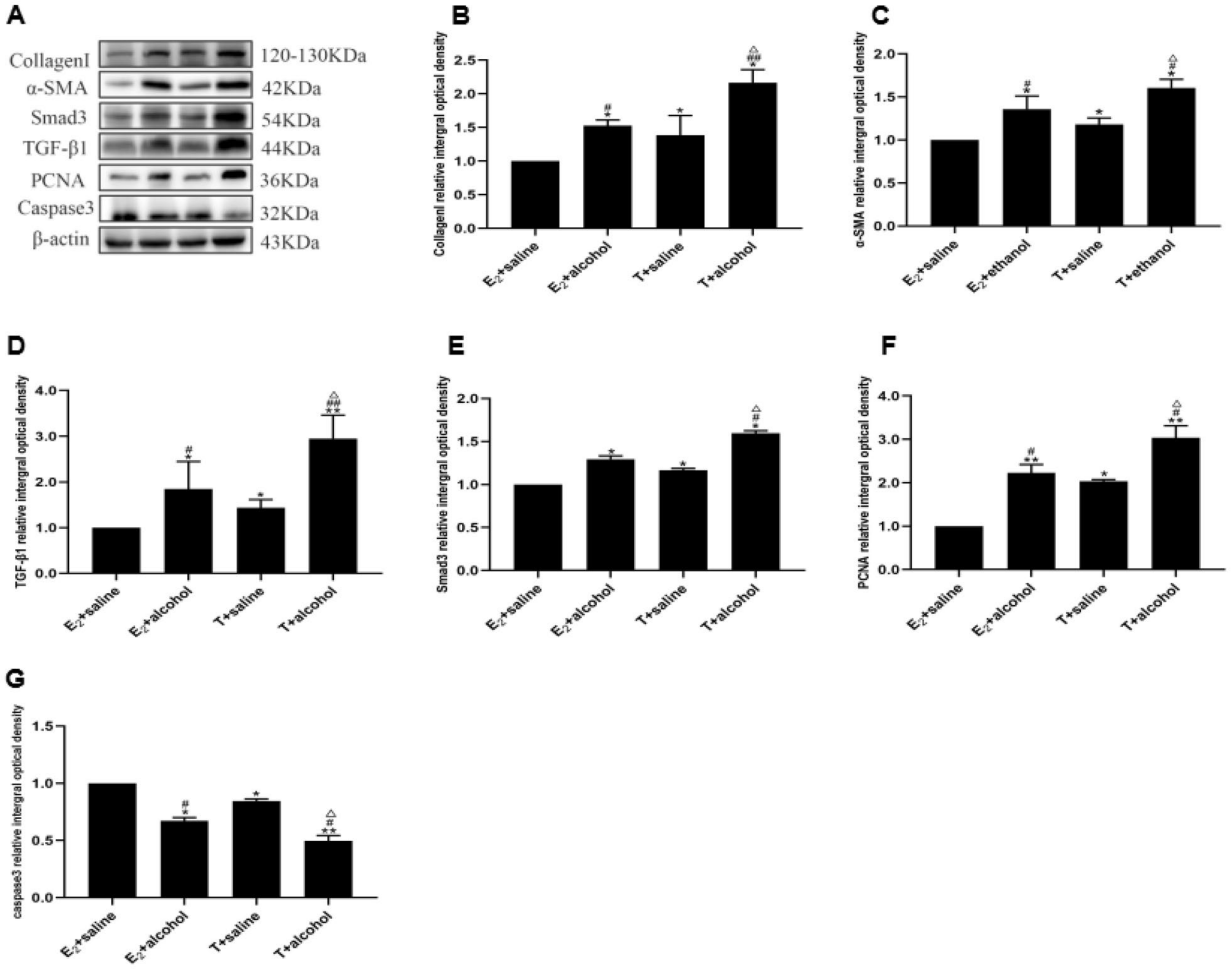
Protein immunoblotting to detect the expression of Collagen-I, α-SMA, TGF-β1, Smad3, PCNA, and Caspase-3 in different groups of cells. **a** Immunoblot bands of Collagen-I, α-SMA, TGF-β1, and Smad3 in different groups of mice; **b** expression changes in Collagen-I; **c** expression changes in α-SMA; **d** expression changes in TGF-β1; **e** expression changes in Smad3; **f** expression changes in PCNA; **g** expression changes in Caspase-3 (compared with the estradiol group, ^*^P < 0.05 or ^**^P < 0.01; compared with the testosterone group, ^#^P < 0.05 or ^##^P < 0.01; compared with the estradiol + ethanol group, ^△^*P* < 0.05 or.^△△^*P* < 0.01). Data are expressed as mean ± SD, *n* = 15

**Fig. 7:**
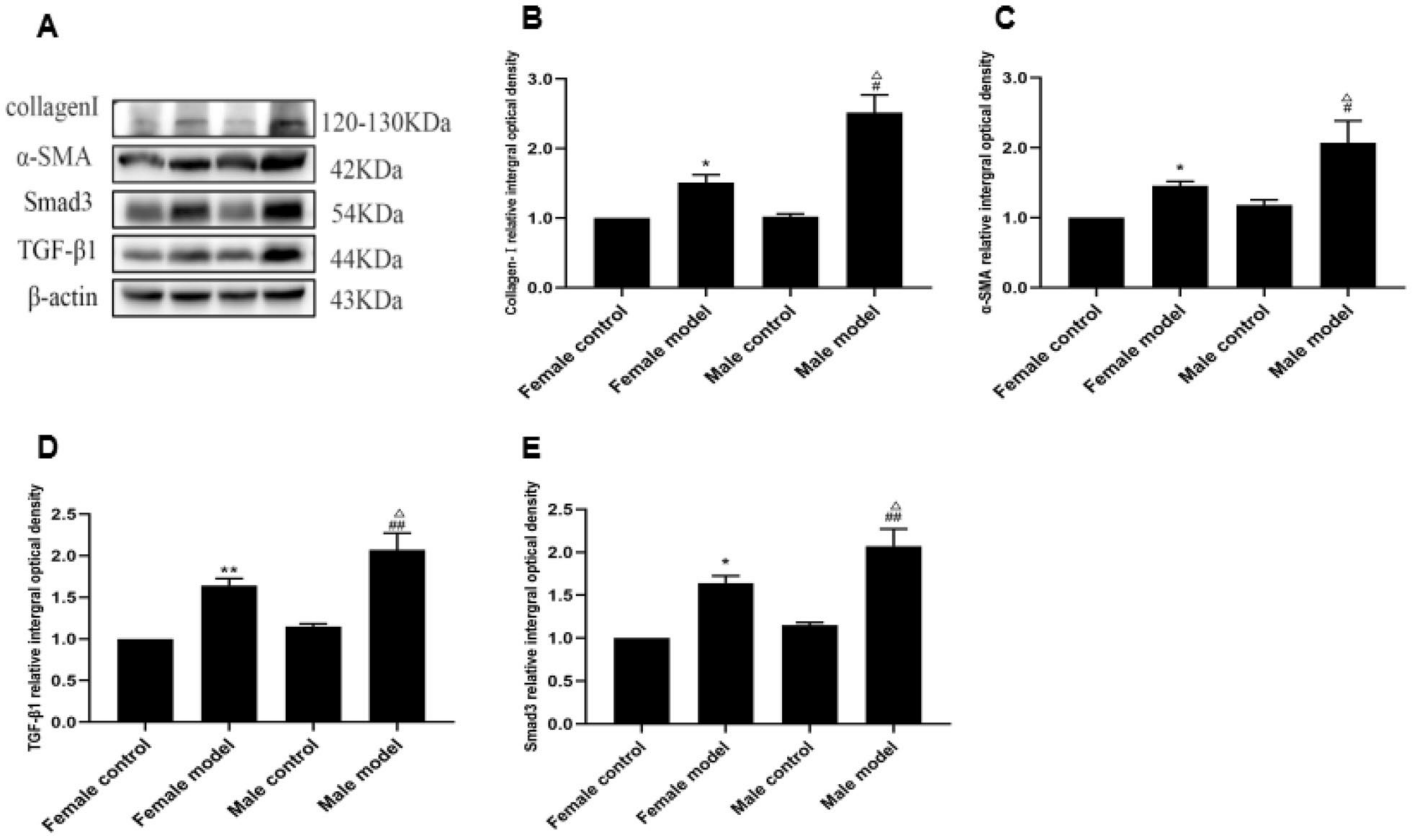
Protein immunoblotting to detect the expression of Collagen-I, α-SMA, TGF-β1, and Smad3 in the liver of different groups of mice. **a** Immunoblot bands of Collagen-I, α-SMA, TGF-β1, and Smad3 in different groups of mice; **b** expression changes in Collagen-I; **c** expression changes in α-SMA; **d** expression changes in TGF-β1; **e** expression changes in Smad3 [compared with the female normal (control) group, ^*^P < 0.05 or ^**^P < 0.01; compared with the male control group, ^#^P < 0.05 or ^##^P < 0.01; compared with the female model group, ^△^*P* < 0.05 or.^△△^*P* < 0.01]. Data are expressed as mean ± SD, *n* = 15

In the in vitro experiments, after induction with estrogen-containing medium and subsequent stimulation with alcohol-containing medium, the testosterone + ethanol group exhibited significantly higher expression levels of Collagen-I, α-SMA, TGF-β1, Smad3, and PCNA and significantly lower expression of Caspase-3 compared with the estradiol + ethanol group (P < 0.05 or P < 0.01). In the in vivo experiment, following gavage with alcoholic liquid feed combined with 31.5% ethanol, the male model group displayed significantly higher expression levels of Collagen-I, α-SMA, TGF-β1, and Smad3 compared with the female model group (*P* < 0.05 or *P* < 0.01). Data are expressed as mean ± SD, *n* = 15 (Figs. 6 and 7).

## Discussion

Liver disease is prevalent globally, with a higher incidence of end-stage liver fibrosis, cirrhosis, and liver cancer in men compared with women, influenced by sex hormones [4]. Clinical findings have revealed that alcoholic liver fibrosis exhibits lower rates in females, with slower progression, and increased survival compared with those in males, possibly due to androgen levels [5]. In a rat model of alcoholic liver fibrosis, androgen-treated desiccated rats experienced more severe fibrosis, pronounced hepatocyte necrosis, and heavier lymphocyte infiltration compared with estrogen-treated desiccated rats, whereas female animals showed significantly less hepatic fibrosis compared with males [6]. Another study showed that administration of varying estrogen doses to male rats with dimethylnitrosamine-induced liver fibrosis inhibited fibrosis, revealing an inverse correlation between estrogen doses and liver fibrosis [7].

The MTT method is used to detect cell survival and growth, providing an indirect measure of living HSC cell count [8]. MTT results revealed that for concentrations in the range 10^−9^ to 10^−7^ mol·L^−1^, E_2_ inhibited HSC growth, with the most significant inhibition observed at 10^−7^ mol·L^−1^. Conversely, T promoted HSC growth, with the most significant promotion observed at 10^−7^ mol·L^−1^.

Flow cytometry results revealed that the apoptosis rate was lower in the testosterone + saline group compared with the estradiol + saline, estradiol + alcohol, and testosterone + alcohol groups. Furthermore, the apoptosis rate was significantly lower in the testosterone + alcohol group compared with the estradiol + saline group, indicating a more pronounced effect of T on HSCs compared with E_2_.

Caspase-3, a key execution molecule mediating apoptosis, and PCNA, a proliferating cell nuclear antigen involved in cell cycle replication and growth, play crucial roles during early apoptosis, Caspase-3 is activated and PCNA activity decreases [9]. Western blot analysis revealed that PCNA expression was increased and Caspase-3 expression was decreased in testosterone + alcohol group compared with estradiol + alcohol group. These findings suggest reduced apoptosis in the testosterone + alcohol group and increased apoptosis in the estradiol + alcohol group. Thus, T and E_2_ inhibit and promote HSC apoptosis, respectively. Moreover, the synergistic effect of T and alcohol enhances HSC activation.

GPT and GOT are biomarkers that reflect the severity of various chronic liver diseases [10]. Hepatocellular injury causes an increase in cell membrane permeability, leading to a rapid surge in the serum activity of both enzymes. Abnormal elevation of GPT and GOT can result in hepatocellular injury and necrosis [11]. TNF-α and IL-6 play crucial roles in acute inflammation. Physical, chemical, and viral factors provoke a significant increase in TNF-α and IL-6 levels [12]. Our in vivo experiments involved the administration of Lieber–DeCarli alcohol liquid diet combined with 31.5% alcohol gavage after molds. At the end of 8 weeks of administration, male mice exhibited significantly higher enzymatic activities of GPT and GOT compared with females. In vitro experiments using an enzyme-linked immunoassay indicated elevated concentrations of TNF-α and IL-6 in the testosterone + saline group compared with the estradiol + saline group. Similarly, the testosterone + alcohol group exhibited increased concentrations of TNF-α and IL-6 compared with the estradiol + alcohol group. Our i*n vivo* experiments further revealed significantly higher concentrations of TNF-α and IL-6 in the male model group compared with the female model group. These findings indicate that male mice exhibit more pronounced hepatocyte injury and inflammatory response following alcohol stimulation compared with females. Moreover, the male model group displayed significantly lower concentrations of androgens and estrogens compared with the female model group in the context of liver fibrosis.

Liver histomorphometry showed that in the female model group, fibrous tissue, and slight hepatocellular enlargement were observed. Conversely, the male model group displayed extensive proliferation of fibrous tissue hepatocytes, along with swelling, enlargement, balloon-like changes, and more pronounced cell necrosis. The HE necrosis score was also significantly higher in the male model group compared with the female model group. Compared with the female model group, the male model group exhibited a significantly higher content of collagen fibers, suggesting a more pronounced degree of fibrosis in male mice after alcohol induction. The male model group displayed a significantly lower glycogen content in liver tissues compared with the female model group, indicating more obvious liver glycogen depletion and greater liver damage in male mice. Moreover, the male model group displayed an increased content of red lipid droplets compared with the female model group, suggesting a more pronounced disorder of lipid metabolism and lipoatrophy in male mice compared with females.

α-SMA serves as a marker for HSC activation [13], whereas Collagen-I is a reliable indicator of successful hepatic fibrosis modeling [14]. In the in vitro experiment, the expression of α-SMA and Collagen-I increased in the testosterone + alcohol group compared with the estradiol + alcohol group, suggesting a more pronounced degree of HSC activation and fibrosis in the former group. Furthermore, the male model group exhibited significantly higher expression of α-SMA and Collagen-I compared with the female model group, indicating a greater propensity for alcohol-induced liver fibrosis in male mice.

The TGF-β1/Smad3-signaling pathway plays a crucial role in liver fibrosis progression [15]. A previous study indicated that the TGF-β1/Smad3-signaling pathway is vital for maintaining normal testicular development and spermatogenesis and is involved in various processes, including sex differentiation, embryonic development, and spermatogenesis [16]. TGF-β1 negatively regulates testicular cell proliferation and development, contributing to the maintenance of normal testicular function and germ cell development [17]. In addition, in mouse ovarian granulosa cells, TGF-β1 enhances E_2_ secretion, increases mRNA levels of the aromatase gene *CYP19A1*, and promotes granulosa cell proliferation. Furthermore, it has been shown that miR-224, which is significantly upregulated in response to TGF-β1 stimulation, stimulates E_2_ secretion and aromatase gene expression in granulosa cells [18]. Our in vitro experiments revealed higher expression of TGF-β1 and Smad3 in the testosterone + alcohol group compared with the estradiol + alcohol group, indicating greater activation of the TGF-β1/Smad3-signaling pathway in the former group. The in vivo experiments also showed significantly increased expression of TGF-β1 and Smad3 in the male model group compared with the female model group, suggesting that alcohol-induced liver fibrosis is more prominent in male mice. These findings underscore the significant gender differences in alcoholic liver fibrosis and their close association with the TGF-β1/Smad3-signaling pathway.

## Conclusion

The degree of fibrosis in alcoholic liver fibrosis exhibited greater prominence in male mice than in females. E_2_ exerted inhibitory effects on HSC growth in females, whereas T promoted HSC growth in males, thereby enhancing HSC activity and subsequently activating the TGF-β1/Smad3-signaling pathway. Consequently, fibrosis levels significantly increased in male mice. These findings serve as a theoretical foundation for the management of alcoholic liver diseases and offer valuable insights for the identification and development of novel therapeutic approaches during treatment strategy formulation.

## Data Availability

All the data and materials are available.

## Abbreviations

ECM: Extracellular matrix
HSC: Hepatic stellate cells
PBST: PBS with Tween
PI: Propanol iodide
